# Machine-Aided Self-diagnostic Prediction Models for Polycystic Ovary Syndrome: Observational Study

**DOI:** 10.2196/29967

**Published:** 2022-03-15

**Authors:** Angela Zigarelli, Ziyang Jia, Hyunsun Lee

**Affiliations:** 1 Department of Mathematics and Statistics University of Massachusetts Amherst Newton, MA United States

**Keywords:** Polycystic Ovary Syndrome (PCOS), prediction, machine learning, self-diagnosis, principal component analysis, clustering, CatBoost, SHAP values, subgroup study

## Abstract

**Background:**

Artificial intelligence and digital health care have substantially advanced to improve and enhance medical diagnosis and treatment during the prolonged period of the COVID-19 global pandemic. In this study, we discuss the development of prediction models for the self-diagnosis of polycystic ovary syndrome (PCOS) using machine learning techniques.

**Objective:**

We aim to develop self-diagnostic prediction models for PCOS in potential patients and clinical providers. For potential patients, the prediction is based only on noninvasive measures such as anthropomorphic measures, symptoms, age, and other lifestyle factors so that the proposed prediction tool can be conveniently used without any laboratory or ultrasound test results. For clinical providers who can access patients’ medical test results, prediction models using all predictor variables can be adopted to help health providers diagnose patients with PCOS. We compare both prediction models using various error metrics. We call the former model the patient model and the latter, the provider model throughout this paper.

**Methods:**

In this retrospective study, a publicly available data set of 541 women’s health information collected from 10 different hospitals in Kerala, India, including PCOS status, was acquired and used for analysis. We adopted the CatBoost method for classification, *K*-fold cross-validation for estimating the performance of models, and SHAP (Shapley Additive Explanations) values to explain the importance of each variable. In our subgroup study, we used *k*-means clustering and Principal Component Analysis to split the data set into 2 distinct BMI subgroups and compared the prediction results as well as the feature importance between the 2 subgroups.

**Results:**

We achieved 81% to 82.5% prediction accuracy of PCOS status without any invasive measures in the patient models and achieved 87.5% to 90.1% prediction accuracy using both noninvasive and invasive predictor variables in the provider models. Among noninvasive measures, variables including acanthosis nigricans, acne, hirsutism, irregular menstrual cycle, length of menstrual cycle, weight gain, fast food consumption, and age were more important in the models. In medical test results, the numbers of follicles in the right and left ovaries and anti-Müllerian hormone were ranked highly in feature importance. We also reported more detailed results in a subgroup study.

**Conclusions:**

The proposed prediction models are ultimately expected to serve as a convenient digital platform with which users can acquire pre- or self-diagnosis and counsel for the risk of PCOS, with or without obtaining medical test results. It will enable women to conveniently access the platform at home without delay before they seek further medical care. Clinical providers can also use the proposed prediction tool to help diagnose PCOS in women.

## Introduction

### Background

There has been substantial advancement in artificial intelligence and machine learning technologies in health care owing to the prolonged COVID-19 pandemic, resulting in improvements and enhancements in medical diagnosis and treatment that were previously impossible or unavailable [1]. Telehealth using remote technologies between medical providers and patients is another emerging trend during the pandemic, and many conditions are diagnosed and managed through telehealth, including polycystic ovary syndrome (PCOS) [2,3]. Diagnosis of PCOS with telehealth is based on a few symptoms such as irregular menstrual cycles, hirsutism, skin problems, and other symptoms caused by an imbalance of androgen hormones [3]. Through our proposed study, integrating the current trends addressed above, we provide a more systematic self-diagnostic tool that allows users to conveniently access and learn the predicted diagnosis result of PCOS without delay before they seek further medical care.

PCOS is the most common endocrine disorder among women of reproductive age, possibly causing infertility. The prevalence of PCOS ranges from 6% to 20%, depending on the population and the diagnostic criteria reported in previous studies [4-6]. Although the cause of this syndrome is not clearly known, there is increasing evidence that PCOS is a complex multigenic disorder with strong epigenetic and environmental influences [4]. According to the Centers for Disease Control and Prevention, overweight women with PCOS may develop serious health problems such as diabetes, gestational diabetes, heart disease, high blood pressure (BP), sleep apnea, and stroke. PCOS is also known to be linked to anxiety and depression [7]. It is important to note that not all women with PCOS experience the same combination or severity of symptoms, which makes early detection more challenging [8].

Although several criteria for PCOS diagnosis have been proposed, the Rotterdam criteria were accepted by the National Institutes of Health, and they have been most commonly adopted for the diagnosis of PCOS [9]. On the basis of the Rotterdam criteria, the diagnosis is made if at least two out of three of the following criteria are met: ovulatory dysfunction (oligo-ovulation or anovulation), higher levels of androgens in the blood, and polycystic ovaries appearing on ultrasound. However, the Rotterdam criteria have been controversial in many studies [9-11].

There are two types of PCOS: lean and obese, each with different biochemical, hormonal, and metabolic profiles [12]. Toosy et al [8] noted that a smaller proportion of women with lean PCOS had a normal or low BMI (≤25 kg/m^2^) and may or may not have symptoms, which makes diagnosis more challenging. In other studies, it has been noted that PCOS is closely associated with obesity and is more prevalent among overweight or obese women than in the general population of women of reproductive age [13-15]. In our proposed study, we incorporate the ideas of lean and obese PCOS types in earlier studies and group the data set into 2 subgroups based on anthropomorphic measures.

### Objectives

Machine learning and deep learning techniques have been widely used to analyze health data and improve diagnostic accuracy and precision, disease treatment, and prevention [16,17]. In particular, feature selection, clustering algorithms, and classification have often been adopted for subgroup studies [18,19]. The goal of this proposed study is to develop a machine-aided self-diagnostic tool that predicts the diagnosis of PCOS with and without any invasive measures, using Principal Component Analysis (PCA), *k*-means clustering algorithm, and CatBoost classifier. The CatBoost method is one of the newer gradient boosting decision tree models, and it was recently used in diabetes prediction in the study by Kumar et al [20]. Our development ultimately enables users, either potential patients or clinical providers, to conveniently access this pre- or self-diagnostic digital platform for PCOS from anywhere. This work is well aligned with emerging artificial intelligence and digital health care [21].

The remainder of this paper is organized as follows. In the *Methods* section, we discuss data preparation, provide an overview of statistical analysis, and explain each machine learning technique used in our analysis. In the *Results* section, we report our findings in a subgroup study and evaluate the performance of our proposed prediction models. In the *Discussion* section, the major results are highlighted, and we conclude this paper.

## Methods

### Subjects and Data Preparation

In this retrospective study, we obtained and analyzed a publicly available data set that was collected from 10 different hospitals across Kerala, India [22]. After data cleaning and removing 15 partly missing or high-leverage data points, the study cohort consisted of 526 women aged between 20 and 48 years, of which 170 (32.3%) were diagnosed with PCOS and 356 (67.7%) were not diagnosed with PCOS. The data set included other health information for each subject, such as anthropomorphic attributes, symptoms, laboratory and ultrasound test results, age, blood type, marital status, pregnancy, abortion history, fast food consumption, and exercise. For later use, we classified and named these variables as *anthropomorphic* variables, *symptom* variables, *test result* variables, and *given* variables in this paper.

The *anthropomorphic* variables include six variables: BMI, height, hip circumference, waist circumference, waist-to-hip ratio, and body weight. The *symptom* variables are self-observable variables. The seven symptom variables are acanthosis nigricans (skin darkening in body folds and creases), acne, hair loss, hirsutism, irregular menstrual cycle, length of menstrual cycle, and weight gain. The *test result* variables are based on blood work and ultrasound or any other medical examination, and they include anti-Müllerian hormone (AMH), the number of antral follicles in the left ovary, the number of antral follicles in the right ovary, average follicle size in the left ovary, average follicle size in the right ovary, diastolic BP, endometrium thickness, follicle-stimulating hormone, follicle-stimulating hormone to luteinizing hormone ratio, glycated hemoglobin levels, human chorionic gonadotropin I, human chorionic gonadotropin II, luteinizing hormone, progesterone, prolactin, pulse rate, random glucose test, respiratory rate, systolic BP, thyrotropin, and vitamin D3. The remaining variables in the data set, other than the status of PCOS and the variables mentioned above, are defined as *given* variables which are age, blood type, years of marriage, abortion history, fast food consumption, pregnancy status, and regular exercise.

### Overview of Statistical Analysis

We first examined the difference in attributes between the PCOS-positive group and the PCOS-negative group as a preliminary investigation. In our analysis, we provided two types of prediction models, with and without invasive health measures. In a model, we used only noninvasive variables, that is, anthropomorphic, symptom and given variables, and called this model the *patient* model. In the other model, we used all variables including noninvasive and invasive variables, and called this model the *provider* model. The purpose of proposing both prediction models was to accommodate various users who may or may not have had access to medical test results.

Another part of the statistical analysis was a subgroup study. There have been several subgroup studies of PCOS diagnosis based on different combinations of the 3 Rotterdam criteria [23,24]. Kar et al [24] characterized and classified phenotypes of PCOS in a large cohort of women into subgroups and compared the data of various metabolic complications of these phenotypes. In our subgroup study, we divided 526 women into 2 subgroups based on anthropomorphic attributes. The motivation for this idea originated from predicting lean PCOS and obese PCOS in a more detailed manner. In the remainder of this section, we explain each machine learning technique and the application of these techniques in our analysis.

### PCA Method

The PCA is a dimension reduction method that is often used when there are highly correlated variables in the data set. It increases interpretability to a certain extent and minimizes data loss by combining correlated variables together to create a new set of uncorrelated yet more representative variables [25,26]. We use PCA to perform feature extraction, where several highly correlated anthropomorphic variables are linearly combined to create new axes, that is, the principal components. For example, body weight and BMI are highly correlated and are consequently highly related to the same principal component. The number of principal components for the analysis can be determined by examining the proportion of variance explained and the elbow rule.

### *K*-Means Clustering

We adopted *k*-means clustering, which is an unsupervised machine learning algorithm that makes inferences using only input variables without referring to known outcomes. It is often used to understand the latent structure within a data set by aggregating data points based on certain similarities. We adopted the *k*-means clustering algorithm and chose the *k* value, the number of clusters, based on the silhouette method and elbow rule [27]. The 2 subgroups were generated by applying the 2-means clustering algorithm based on the first five principal components, PC1, PC2, PC3, PC4, and PC5, which were created based on the anthropomorphic attributes. Therefore, the resulting 2 subgroups were very distinct anthropomorphically.

### CatBoost Classification and Cross-Validation

We modeled our classifier using the CatBoost model, which is a gradient boosting decision tree-based classifier. The CatBoost model was introduced by Yandex in 2017 and is known to be more accurate for categorical variables than other well-known gradient boosting algorithms such as XGBoost (The XGBoost Contributors), LightGBM (Microsoft Corporation), and GBM (H2O) [28].

To prevent the classification model from overfitting or underfitting, we used stratified *K*-fold cross-validation to train and test the classification models and evaluate the model performance. We first randomly divided the sample into stratified *K*-folds where the folds were formed by preserving the percentage of PCOS-positive in the sample. We used 1-fold as a validation (hold out) set and the remaining (*K*-1) folds for model training.

In the patient model, we used only noninvasive variables (anthropomorphic, symptom, and given variables) for classification. This prediction tool can be used by potential patients or other users who do not have medical testing available. As we do not use the complete set of predictor variables in this patient model, lower accuracy is unavoidable. The provider model uses all predictor variables. This model is expected to aid clinical providers or other users who have access to the patients’ medical test results for the prediagnosis or self-diagnosis of PCOS status with higher accuracy. A subgroup study was applied to both models, as summarized in Table 1. We used 5-fold cross-validation with 3 iterations for the entire data set analysis (with a sample size of 526) and 3-fold cross-validation with 5 iterations for the subgroup analysis (with the sample sizes of 287 for Subgroup 1 and 239 for Subgroup 2) to achieve a comparable number of subjects in each fold and averaged the errors over the total 15 folds in both analyses for comparison.

To evaluate the performance of the models, we used four error metrics: accuracy, sensitivity, precision, and F_1_ score. Accuracy is the most intuitive and common performance measure, and it is the ratio of correctly predicted observations to the total observations. Sensitivity is the ratio of correctly predicted positive observations to all true positive observations in the class. Precision is the ratio of correctly predicted positive observations to the total number of predicted positive observations. Finally, the F_1_ score is the weighted average of the sensitivity and precision. Therefore, it considers both false positives and false negatives. The F_1_ score is more useful than the accuracy when there is an uneven class distribution.

**Table 1:** Proposed models and the difference in method.

			Model description		Predictor variables
**Patient model**					
	Subgroup model	Different model for each subgroup		Only noninvasive variables	
	One model to all	One model for the entire data set		Only noninvasive variables	
**Provider model**					
	Subgroup model	Different model for each subgroup		All variables	
	One model to all	One model for the entire data set		All variables	

### Shapley Additive Explanations Values and Feature Importance

The SHAP (Shapley Additive Explanations) values proposed by Lundberg and Lee [29] were used to examine how a single feature affects the output of the model by measuring the change in log odds. The SHAP values can be considered as credit values that are optimally allocated with local explanations using the classic Shapley values in game theory. The SHAP values were first calculated for each variable for each subject in the entire data set and averaged over the sample subjects. SHAP values farther away from 0 have a greater impact on the model output, either positively or negatively. The corresponding total variable importance was calculated by averaging the absolute SHAP values for the sample subjects. This variable importance depicts the magnitude of the total impact of each variable on the model output.

**Figure 1:**
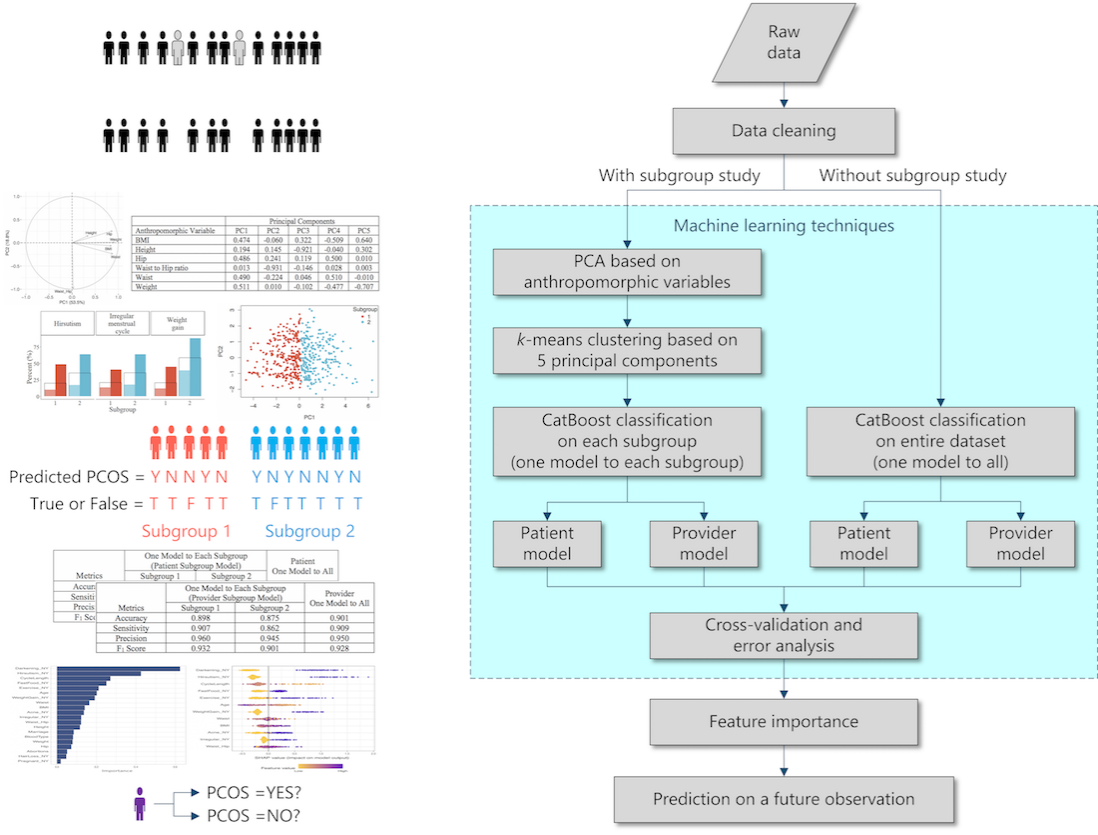
Illustration of the subgroup study and the flowchart of data analysis. PCA: Principal Component Analysis; PCOS: polycystic ovary syndrome.

All statistical analyses were performed using R statistical software (version 4.0.3; R Foundation for Statistical Computing). Figure 1 illustrates the subgroup study and summarizes the statistical procedures in a flowchart.

## Results

### Attributes

**Figure 2:**
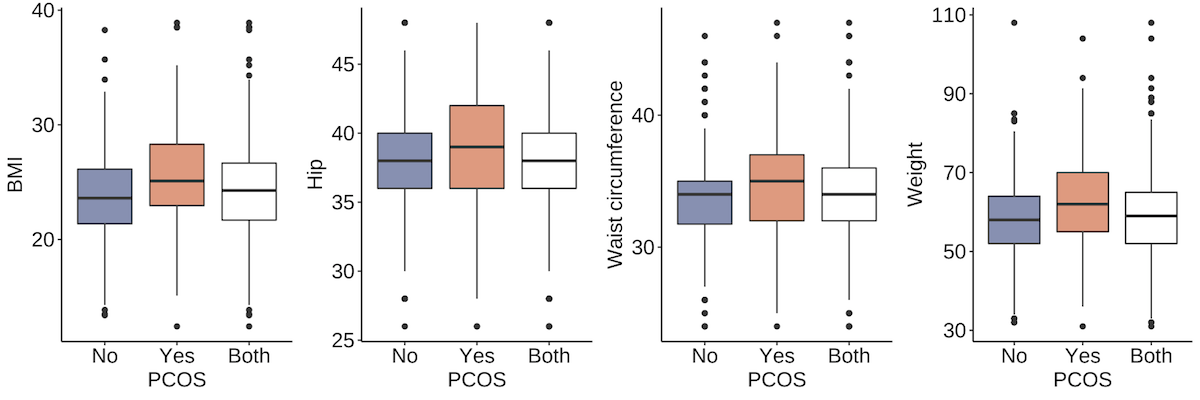
Anthropomorphic variable comparison between the polycystic ovary syndrome (PCOS) positive group and the PCOS-negative group with *P*<.001.

**Figure 3:**
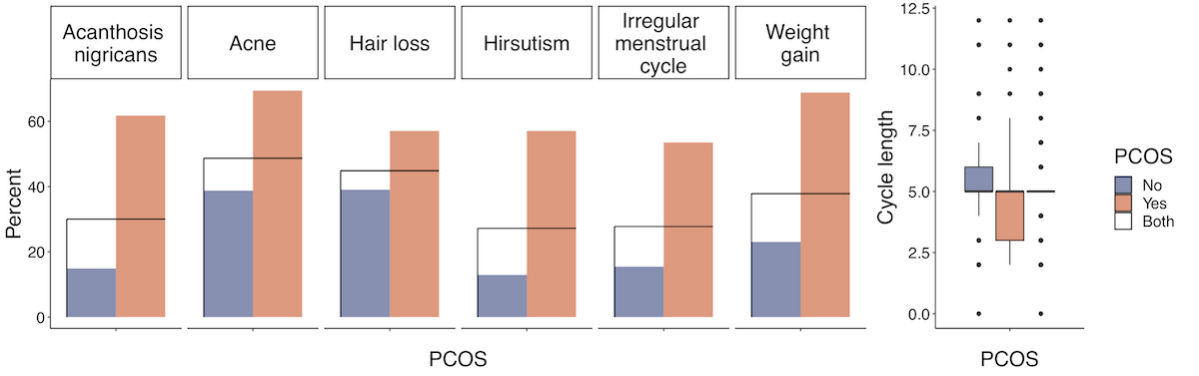
Symptom variable comparison between the polycystic ovary syndrome (PCOS) positive group and the PCOS-negative group with *P*<.001.

**Figure 4:**
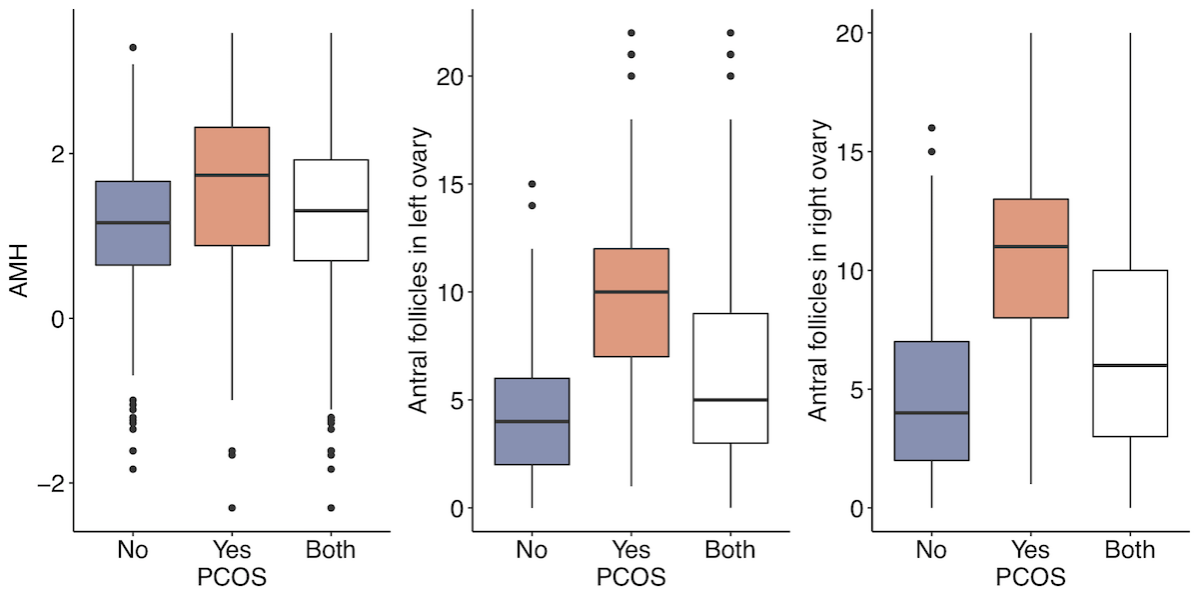
Test result variable comparison between the polycystic ovary syndrome (PCOS) positive group and the PCOS-negative group with *P*<.001. AMH: anti-Müllerian hormone.

**Figure 5:**
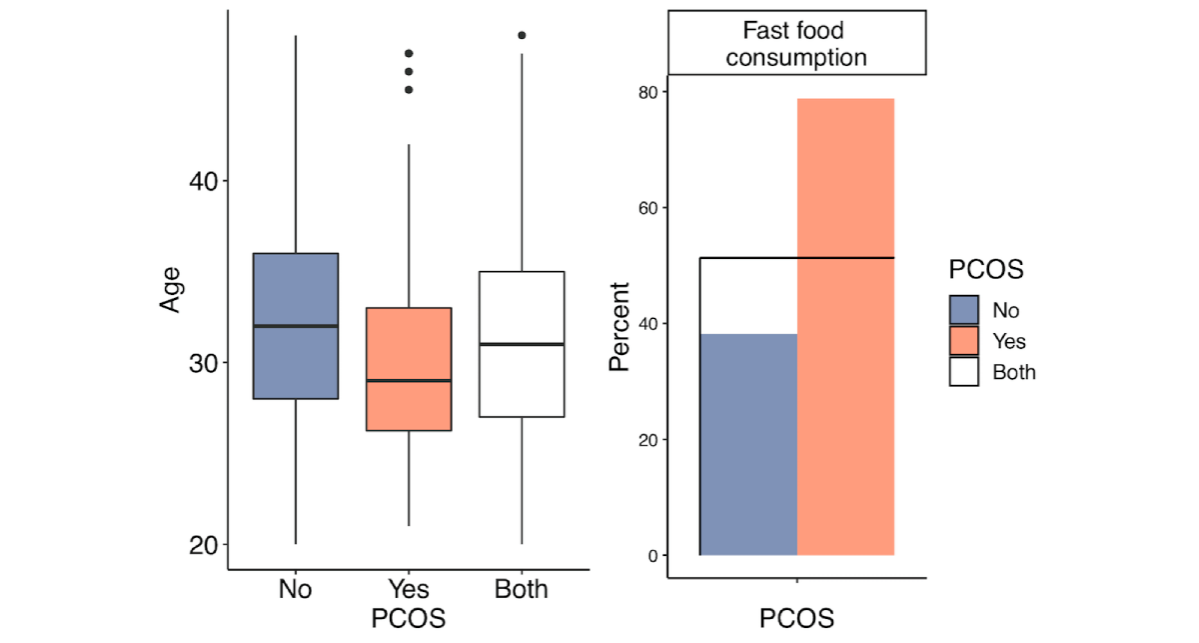
Given variable comparison between the polycystic ovary syndrome (PCOS) positive group and the PCOS-negative group with *P*<.001.

We first examined the differences between the PCOS-positive group and the PCOS-negative group to provide an overview of the data set. We used the Anderson-Darling test and found that none of the quantitative variables were normally distributed; therefore, we summarized those variables with median and IQR. For categorical variables, we used percentage. The Wilcoxon test was used to compare the medians for quantitative attributes, and the chi-square test was used to compare the proportions of qualitative attributes between the groups. The corresponding *P* values are presented in the last column of Table 2. Highly significant differences with *P*<.001 in variables were found in BMI, hip circumference, waist circumference, and body weight among the anthropomorphic variables, all symptom variables, AMH, number of antral follicles in the left ovary and number of antral follicles in the right ovary among the test result variables, and age and fast food consumption among the given variables. These results are consistent with those of the previous studies. Anthropomorphic variables have often been linked to PCOS [30]. Symptoms and AMH have also been reported as important markers for the diagnosis of PCOS [31]. As the number of antral follicles in the left ovary and number of antral follicles in the right ovary are used in the diagnosis of PCOS, the significant differences in these variables between the 2 groups were significant. Finally, age and diet habits were found to be important factors for PCOS [32,33]. Figures 2-5 display the group comparison of these variables in detail.

**Table 2:** Anthropomorphic, symptom, test result, and given variables. Comparison between the PCOS^a^-positive group and the PCOS-negative group with *P* values (N=526).

				All women (N=526)		PCOS-positive size (n=170)		PCOS-negative size (n=356)		*P* value^b^	
**Anthropomorphic variables, median (IQR)**											
	BMI		24.27 (21.69 to 26.66)		25.1 (26.25 to 33)		23.61 (21.37 to 26.13)		<.001		
	Height (cm)		156 (152 to 160)		158 (152 to 161)		156 (152 to 160)		.12		
	Hip (inches)		38 (36 to 40)		39 (36 to 42)		38 (36 to 40)		<.001		
	Waist circumference (inches)		34 (52 to 65)		35 (32 to 37)		34 (31.75 to 35)		<.001		
	Waist-to-hip ratio		0.89 (0.88 to 0.93)		0.89 (0.86 to 0.93)		0.89 (0.86 to 0.93)		.91		
	Weight (kg)		59 (52 to 65)		62 (55 to 70)		58 (52 to 64)		<.001		
**Symptom variables**											
	Acanthosis nigricans (%)		158 (30)		105 (61.8)		53 (14.9)		<.001		
	Acne (%)		256 (48.7)		118 (69.4)		138 (38.8)		<.001		
	Hair loss (%)		236 (44.9)		97 (57.1)		139 (39)		<.001		
	Hirsutism (%)		143 (27.2)		22 (12.9)		46 (12.9)		<.001		
	Irregular menstrual cycle (%)		146 (27.8)		91 (53.5)		55 (15.5)		<.001		
	Length of menstrual cycle (days), median (IQR)		5 (5 to 5)		5 (3 to 5)		5 (5 to 6)		<.001		
	Weight gain (%)		199 (37.8)		117 (68.8)		82 (23)		<.001		
**Test result variables, median (IQR)**											
	AMH^c,d^ (ng/mL)		1.31 (0.70 to 1.92)		1.74 (0.88 to 2.32)		1.16 (0.65 to 1.66)		<.001		
	Antral follicles in left ovary		5 (3 to 9)		10 (7 to 12)		4 (2 to 6)		<.001		
	Antral follicles in right ovary		6 (3 to 10)		11 (8 to 13)		4 (2 to 7)		<.001		
	Average follicle size left ovary (mm)		15 (13 to 18)		16 (14 to 18)		15 (13 to 18)		.02		
	Average follicle size right ovary (mm)		16 (13 to 18)		16 (14 to 18)		15.5 (13 to 18)		.046		
	Diastolic BP (mm Hg)		80 (70 to 80)		80 (70 to 80)		80 (70 to 80)		.69		
	Endometrium thickness (mm)		8.5 (7 to 9.8)		8.9 (7.6 to 10)		8.3 (7 to 9.5)		.006		
	FSH^c,e^ (mIU/mL)		1.58 (1.2 to 1.85)		1.51 (1.17 to 1.75)		1.61 (1.25 to 1.88)		.007		
	FSH to LH^f^ ratio^c^		0.77 (0.35 to 1.35)		0.69 (0.07 to 1.14)		0.86 (0.44 to 1.41)		.002		
	Glycated hemoglobin level (g/100 ml)		11 (10.5 to 11.7)		11.05 (10.7 to 11.9)		11 (10.5 to 11.5)		.03		
	HCG^g^ I^c^ (mIU/mL)		2.96 (0.688 to 5.7)		4.25 (0.69 to 5.85)		2.68 (0.69 to 5.62)		.10		
	HCG II^c^ (mIU/mL)		0.69 (0.688 to 4.61)		0.69 (0.69 to 4.63)		0.69 (0.69 to 4.59)		.94		
	LH (mIU/mL)		2.25 (1.03 to 3.68)		2.23 (1.03 to 4.31)		2.3 (1.03 to 3.6)		.25		
	PRG^c,h^ (ng/mL)		−1.14 (−1.39 to −0.78)		−1.14 (−1.39 to −0.84)		−1.17 (−1.39 to −0.78)		.54		
	PRL^c,i^ (ng/mL)		3.09 (2.68 to 3.39)		3.13 (2.64 to 3.41)		3.06 (2.69 to 3.39)		.73		
	Pulse rate (beats/min)		72 (72 to 74)		72.5 (72 to 74)		72 (72 to 74)		.002		
	Random glucose test (mg/100 mL)		100 (92 to 107)		100 (92 to 107)		97.5 (92 to 108)		.32		
	Respiratory rate (breaths/min)		18 (18 to 20)		20 (18 to 20)		18 (18 to 20)		.28		
	Systolic BP^j^ (mm Hg)		110 (110 to 120)		110 (110 to 120)		110 (110 to 120)		.73		
	Thyrotropin^c^ (mIU/L)		0.82 (0.39 to 1.27)		0.84 (0.39 to 1.26)		0.78 (0.39 to 1.27)		.69		
	Vitamin D3 (ng/mL)		25.95 (20.73 to 34.48)		25.38 (19.3 to 33.58)		26.3 (21.28 to 35.8)		.19		
**Given variables**											
	Age (years), median (IQR)		31 (27 to 35)		29 (26.25 to 33)		32 (28 to 36)		<.001		
	**Blood type (%)**										.97
		A+	105 (20)		33 (19.4)		72 (20.2)				
		A−	13 (2.5)		4 (2.4)		9 (2.5)				
		B+	130 (24.7)		40 (23.5)		90 (25.3)				
		B−	16 (3)		6 (3.5)		10 (2.8)				
		O+	200 (38)		63 (37.1)		137 (38.5)				
		O−	19 (3.6)		8 (4.7)		11 (3.1)				
		AB+	41 (7.8)		15 (8.8)		26 (7.3)				
		AB-	2 (0.4)		1 (0.6)		1 (0.3)				
	Married (years), median (IQR)		7 (4 to 10)		6 (3 to 9)		7 (4 to 11)		.001		
	**Number of abortions** **(%)**										.61
		0	425 (80.8)		141 (82.9)		284 (79.8)				
		1	68 (12.9)		21 (12.4)		47 (13.2)				
		2 and above	33 (6.3)		8 (4.7)		25 (7)				
	Fast food consumption (%)		270 (51.3)		134 (78.8)		136 (38.2)		<.001		
	Pregnant (%)		204 (38.8)		63 (37.1)		141 (39.6)		.64		
	Regular exercise (%)		127 (24.1)		48 (28.2)		79 (22.2)		.16		

^a^PCOS: polycystic ovary syndrome.

^b^*P* values were calculated by the Wilcoxon test for median comparison and chi-square test for proportion comparison.

^c^Log-transformed variables.

^d^AMH: anti-Müllerian hormone.

^e^FSH: follicle-stimulating hormone.

^f^LH: luteinizing hormone.

^g^HCG: human chorionic gonadotropin.

^h^PRG: progrestrone.

^i^PRL: prolactin.

^j^BP: blood pressure.

### Feature Extraction and Clustering

**Figure 6:**
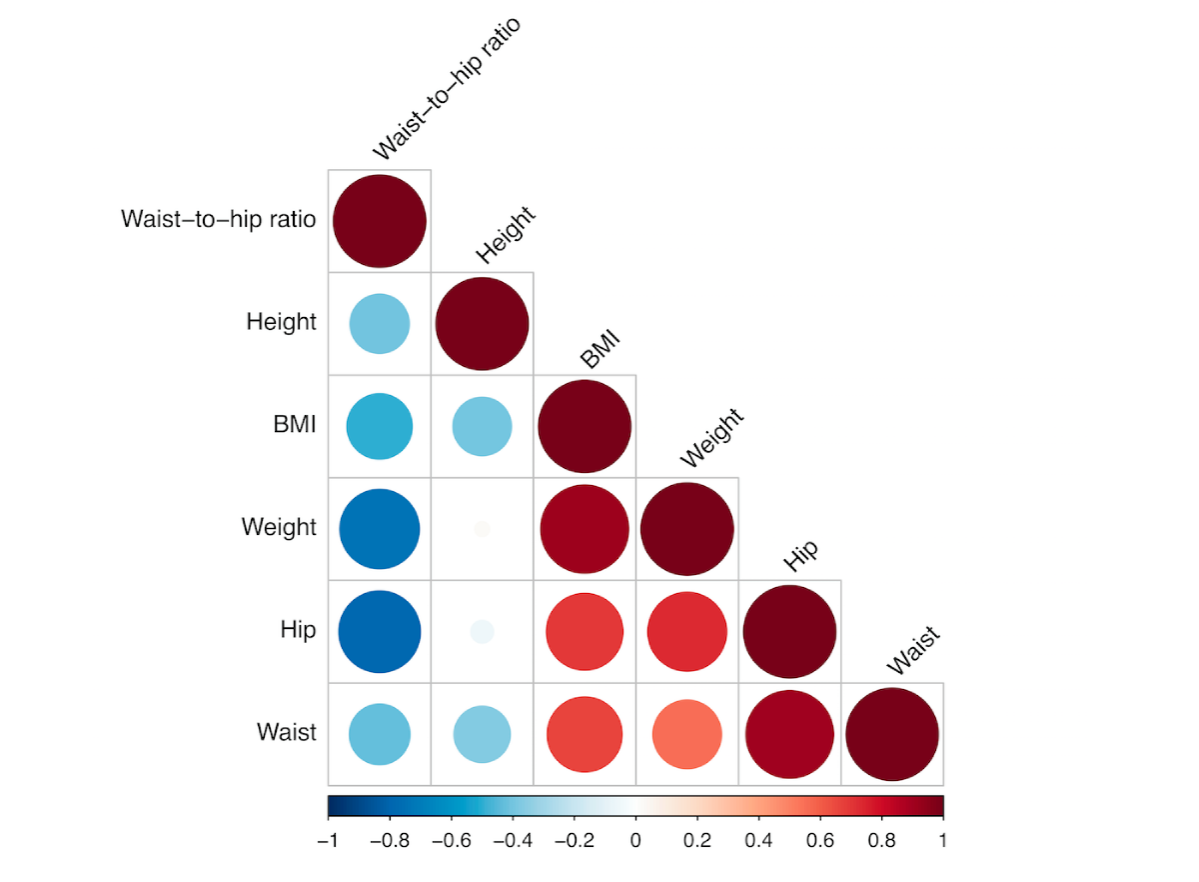
Correlation between the anthropomorphic variables.

**Figure 7:**
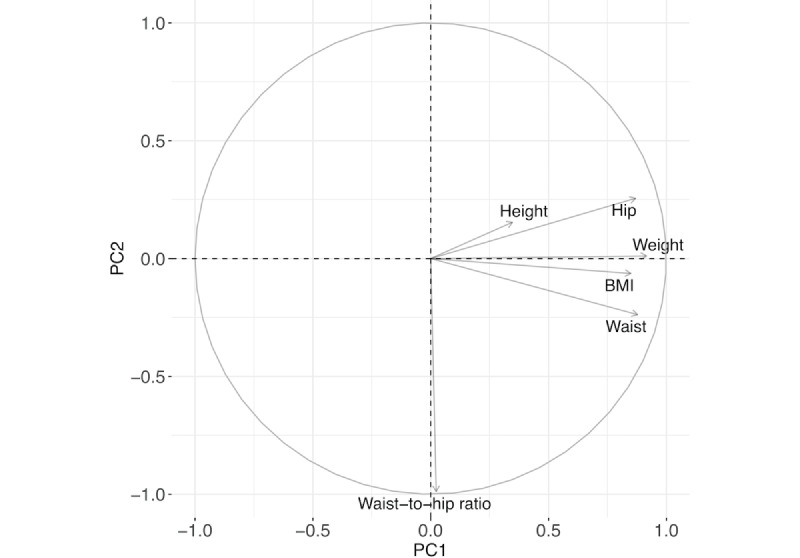
Principal Component Analysis. Biplot of PC1 and PC2 based on the anthropomorphic attributes.

**Figure 8:**
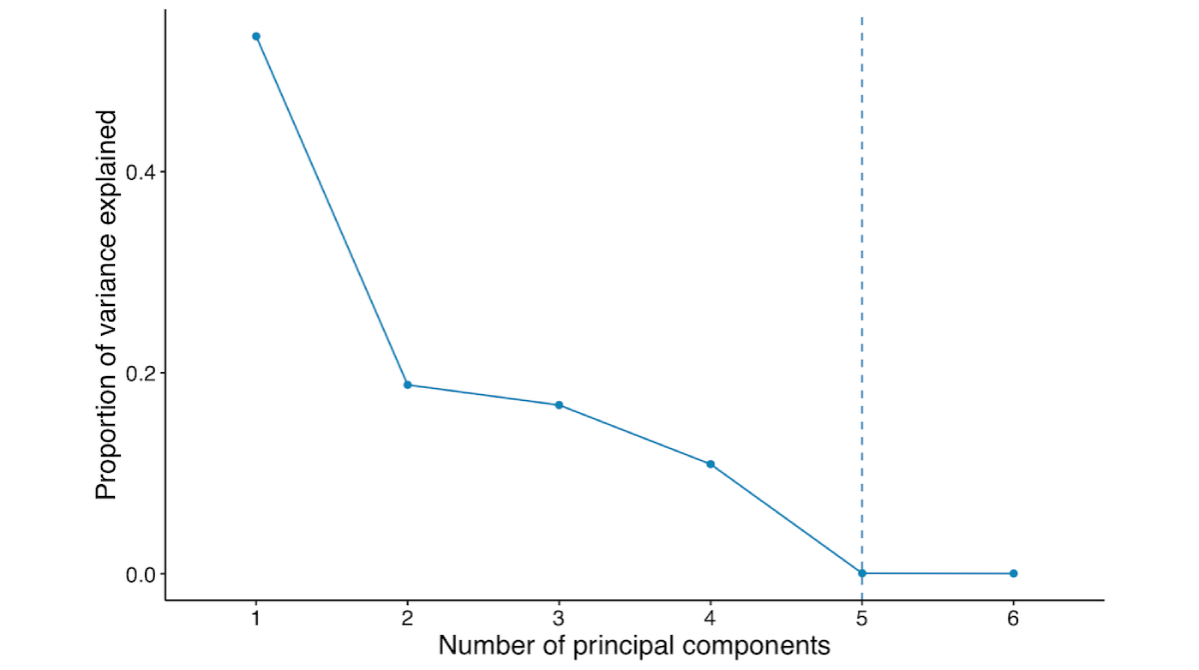
The optimal number of principal components to be used based on the proportion of variance explained.

For the subgroup study, we used the first 5 principal components constructed based on the anthropomorphic attributes and then applied *k*-means clustering using the 5 principal components to classify the 526 women into 2 subgroups. Figure 6 shows the correlation among the anthropomorphic variables. BMI, hip circumference, waist circumference, and body weight are highly and positively correlated, and height and waist-to-hip ratio are negatively correlated to the rest of variables. This can be also observed in PCA. The biplot in Figure 7 and Table 3 explain how each anthropomorphic variable contributes to the individual principal components. For example, as BMI, hip circumference, waist circumference, and body weight are closely correlated as depicted in Figure 6, these variables largely contribute to the first principal component (the horizontal axis, PC1) with high loadings in Figure 7 and Table 3. This newly constructed PC1 accounts for or *explains* 53.5% of the overall variability in Figure 7, and it is the first principal axis in the direction where the data points vary the most. The waist-to-hip ratio variable highly contributes to the second principal component (the vertical axis, PC2) with the second largest proportion (15.8%) of variance explained in Figure 7 and Table 3. The number of principal components is chosen based on the amount of variance explained by using the principal components. In Figure 8, we observe that the proportion of variance explained drops at 5 principal components and we choose the first 5 principal components for clustering in the next step.

**Figure 9:**
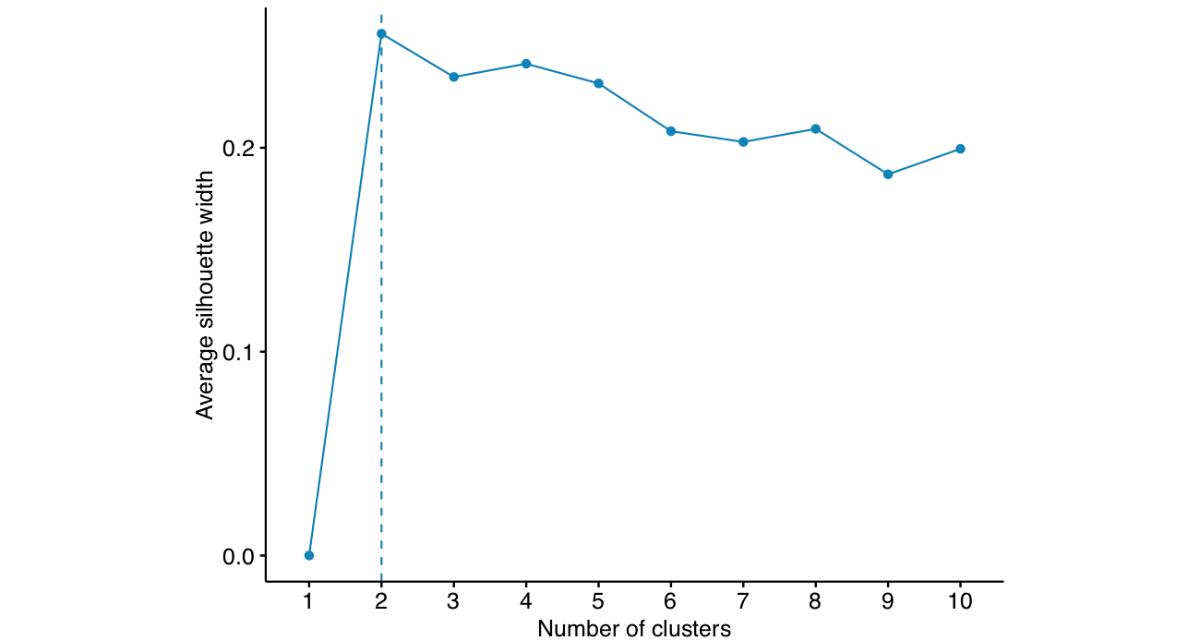
Silhouette method to determine the optimal number of clusters.

**Figure 10:**
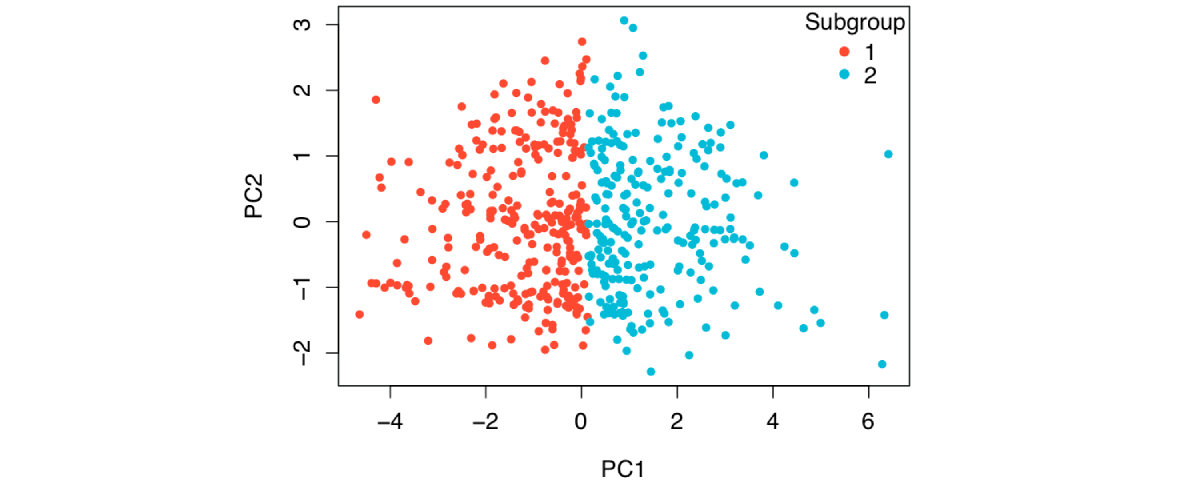
Subgroups on the PC1-PC2 plane after applying *k*-means clustering using the first 5 principal components based on the anthropomorphic attributes.

For *k*-means clustering, the optimal number *(k*) is determined based on the silhouette method and the elbow rule in Figure 9, which shows the average silhouette width versus the number of clusters. As indicated in Figure 9, we chose *k*=2, where the curve shows a sharp kink (the elbow rule). The motivation of the 2-subgroup study also originated from the idea of predicting lean PCOS and obese PCOS in a more detailed manner. In Figure 10, the 2 subgroups are shown in the PC1-PC2 plane. Subgroup 1 included 287 subjects with 76 PCOS-positive cases and subgroup 2 had 239 subjects with 94 PCOS-positive cases. The mean and SD of BMI for Subgroup 1 were 21.862 kg/m^2^ and 2.921 kg/m^2^, and those for Group 2 were 27.246 kg/m^2^ and 3.183 kg/m^2^, respectively. This clustering provides a similar yet more structured subgrouping to the lean and obese PCOS groups, where the lean PCOS group had a BMI <25 kg/m^2^ and the obese PCOS group had a BMI of ≥25 kg/m^2^ [34].

**Table 3:** Variable loadings^a^ related to each principal component.

Anthropomorphic variable	Principal components				
	PC1	PC2	PC3	PC4	PC5
BMI	0.474	−0.060	0.322	−0.509	0.640
Height	0.194	0.145	−0.921	−0.040	0.302
Hip	0.486	0.241	0.119	0.500	0.010
Waist-to-hip ratio	0.013	−0.931	−0.146	0.028	0.003
Waist	0.490	−0.224	0.046	0.510	−0.010
Weight	0.511	0.010	−0.102	−0.477	−0.707

^a^Loading denotes the contribution of the variable to each principal component. Higher absolute value indicates more contribution to the corresponding principal component.

**Figure 11:**
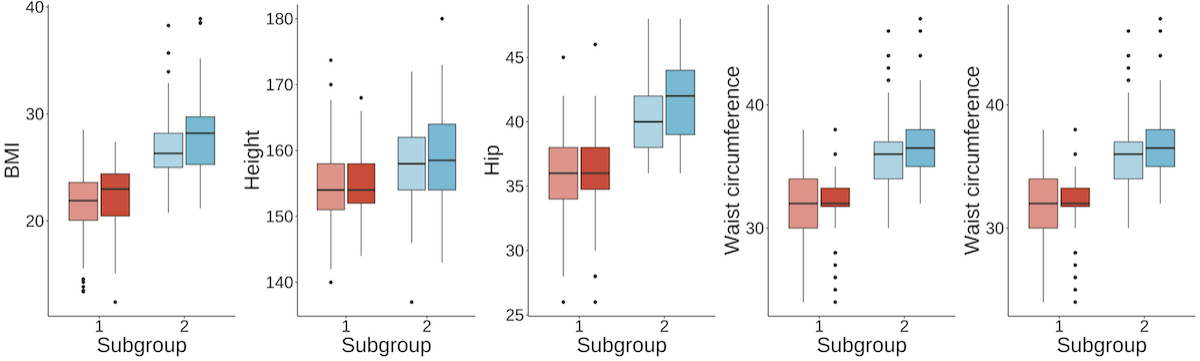
Anthropomorphic variable comparison between subgroup 1 and subgroup 2 with *P*<.001.

**Figure 12:**
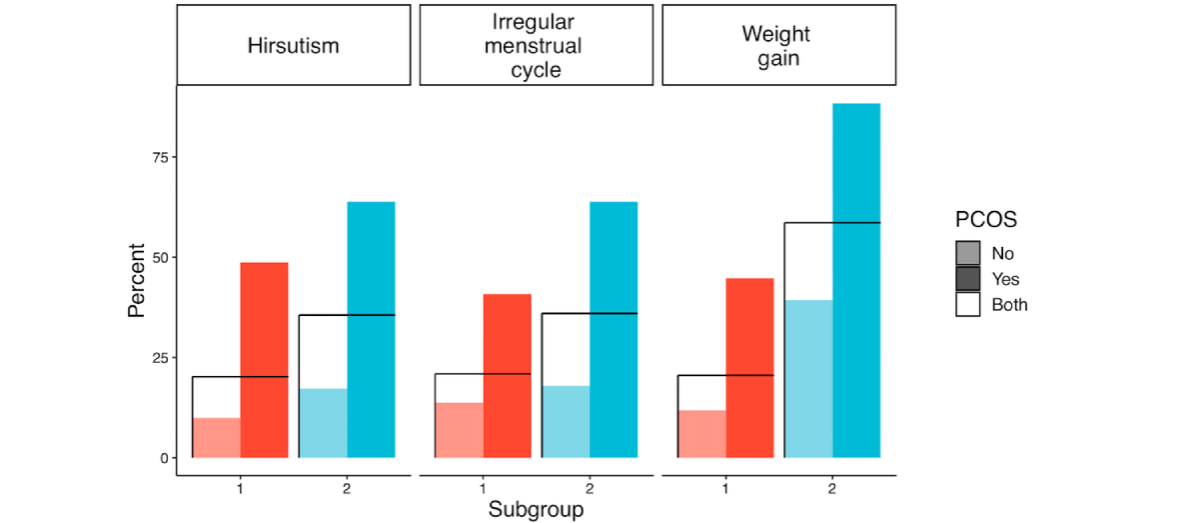
Symptom variable comparison between subgroup 1 and subgroup 2 with *P*<.001. PCOS: polycystic ovary syndrome.

In Table 4, these 2 subgroups are compared in great detail. The same statistical tests used in Table 2 were used for this comparison. These subgroups were generated by using the clustering algorithm based on the first 5 principal components of the anthropomorphic variables as discussed above. Consequently, there were significant differences in all anthropomorphic variables except waist-to-hip ratio. Otherwise, there were highly significant differences with *P*<.001 in hirsutism, irregular menstrual cycle, and weight gain among the symptom variables. We visually compare these variables in Figures 11 and 12.

**Table 4:** Characteristics of 2 subgroups classified by *k*-means clustering based on the 5 principal components of the anthropomorphic variables.

				Subgroup 1; n=287, PCOS^a^-positive, n=76		Subgroup 2; n=239, PCOS-positive, n=94		*P* value^b^	
**Anthropomorphic variables, median (IQR)**									
	BMI (kg/m^2^)			22.15 (20.29 to 23.9)		26.75 (25.1 to 28.98)		<.001	
	Height (cm)			154 (152 to 158)		158 (154 to 1620)		<.001	
	Hip (in)			36 (34 to 38)		40 (39 to 42)		<.001	
	Waist circumference (in)			32 (30 to 34)		36 (35 to 38)		<.001	
	Waist-to-hip ratio			0.89 (0.85 to 0.93)		0.9 (0.86 to 0.93)		.24	
	Weight (kg)			53 (50 to 56)		66 (62 to 72.15)		<.001	
**Symptom variables**									
	Acanthosis nigricans (%)			76 (26.5)		82 (34.3)		.06	
	Acne (%)			141 (49.1)		115 (48.1)		.89	
	Hair loss (%)			123 (42.9)		113 (47.3)		.35	
	Hirsutism (%)			58 (20.2)		85 (35.6)		<.001	
	Irregular menstrual cycle (%)			60 (20.9)		137 (57.2)		<.001	
	Length of menstrual cycle (days), median (IQR)			5 (5 to 5)		5 (4 to 6)		.73	
	Weight gain (%)			59 (20.6)		140 (58.6)		<.001	
**Test result variables, median (IQR)**									
	AMH^c,d^ (ng/mL)			1.34 (0.71 to 1.92)		1.29 (0.69 to 1.92)		.92	
	Antral follicles in left ovary			5 (3 to 8)		6 (3 to 9)		.06	
	Antral follicles in right ovary			5 (3 to 9)		7 (3 to 10)		.06	
	Average follicle size left ovary (mm)			15 (13.5 to 18)		15 (13 to 18)		.27	
	Average follicle size right ovary (mm)			16 (13 to 18)		16 (13 to 18)		.28	
	Diastolic BP^f^ (mm Hg)			80 (70 to 80)		80 (80 to 80)		.02	
	Endometrium thickness (mm)			8.5 (7 to 10)		8.5 (7 to 9.6)		.39	
	FSH^c,f^ (mIU/mL)			1.6 (1.18 to 1.87)		1.53 (1.21 to 1.83)		.34	
	FSH to LH^g^ ratio^c^			0.73 (0.38 to 1.38)		0.67 (0.29 to 1.35)		.93	
	Glycated hemoglobin (g/100 ml)			11 (10.6 to 11.75)		11 (10.5 to 11.7)		.69	
	HCG I^c,h^ (mIU/mL)			3.77 (0.69 to 5.91)		2.3 (0.69 to 5.44)		.02	
	HCG II^c^ (minus/mL)			0.69 (0.69 to 4.51)		0.69 (0.69 to 4.61)		.89	
	LH (maul/mL)			2.34 (1.04 to 3.84)		2.15 (1.03 to 3.46)		.51	
	PRG^c,i^ (ng/mL)			−1.2 (−1.39 to −0.8)		−1.11 (−1.39 to −0.78)		.18	
	PRL^c,j^ (ng/mL)			3.14 (2.75 to 3.14)		3 (2.62 to 3.36)		.02	
	Pulse rate (beats/min)			72 (72 to 74)		72 (72 to 74)		.61	
	Random glucose test (mg/100 mL)			98 (91.5 to 106)		100 (92 to 108)		.17	
	Respiratory rate (breaths/min)			18 (18 to 20)		18 (18 to 20)		.79	
	Systolic BP (mm Hg)			110 (110 to 120)		120 (110 to 120)		.90	
	Thyrotropin^c^ (mIU/L)			0.79 (0.37 to 1.29)		0.83 (0.41 to 1.22)		.98	
	Vitamin D3 (ng/mL)			26 (21.26 to 34.3)		25.69 (20.05 to 34.9)		.56	
**Given variables**									
	Age (years), median (IQR)			31 (28 to 35)		31 (27 to 35)		.33	
	**Blood type (%)**								.007
		A+	59 (20.6)		46 (19.3)				
		A−	6 (2.1)		7 (2.9)				
		B+	79 (27.5)		51 (21.3)				
		B−	12 (4.2)		4 (1.7)				
		O+	99 (34.5)		101 (42.3)				
		O−	5 (1.7)		14 (5.9)				
		AB+	27 (9.4)		14 (5.9)				
		AB−	0 (0)		2 (0.8)				
	Married (years), median (IQR)			7 (4 to 10)		7 (4 to 10)		.97	
	**Number of abortions (%)**								.31
		0	239 (83.3)		186 (77.8)				
		1	35 (12.2)		33 (13.8)				
		2 and above	13 (4.5)		20 (8.4)				
	Fast food consumption (%)			132 (46)		138 (57.7)		.009	
	Pregnant (%)			117 (40.8)		87 (36.4)		.35	
	Regular exercise (%)			83 (28.9)		44 (18.4)		.007	

^a^PCOS: polycystic ovary syndrome.

^b^*P* values were calculated by the Wilcoxon test for median comparison and chi-square test for proportion comparison.

^c^Log-transformed variables.

^d^AMH: anti-Müllerian hormone.

^e^BP: blood pressure.

^f^FSH: follicle-stimulating hormone.

^g^LH: luteinizing hormone.

^h^HCG: human chorionic gonadotropin.

^i^PRG: progrestrone.

^j^PRL: prolactin.

### Performance Evaluation

The prediction results from the 4 different models in Table 1 are compared in Tables 5 and 6. We used four different error metrics: accuracy, sensitivity, precision, and F_1_ score. Table 5 shows the results of the patient models with and without using subgroups. As expected, the subgroup models had overall lower performance in predicting PCOS status. With subgroups, the prediction in the lower BMI group (subgroup 1) was slightly higher than that of the other subgroup. This might be because women with higher BMI are more likely to have other complications [14,15], causing more difficulty in predicting the status of PCOS. In Table 6, we present a comparison of the error metrics for the provider models with and without subgroups. In the provider models, we predict the status of PCOS using the complete set of predictor variables, including the noninvasive variables as well as the test result variables. Therefore, the model prediction is overall better than the patient models, where we use only noninvasive predictors.

**Table 5:** Summary of averaged accuracy, sensitivity, precision, and F_1_ score for one model to each subgroup (with subgroups) versus one model to all (without subgroups) in the patient model, using only noninvasive predictor variables in the models.

Metrics	Patient model with subgroup		Patient model without subgroup
	Subgroup 1	Subgroup 2	
Accuracy	0.815	0.810	0.825
Sensitivity	0.837	0.827	0.851
Precision	0.931	0.870	0.900
F_1_ score	0.880	0.846	0.874

**Table 6:** Summary of averaged accuracy, sensitivity, precision, and F_1_ score for one model to each subgroup (with subgroups) versus one model to all (without subgroups) in the provider model, using all predictor variables in the models.

Metrics	Provider model with subgroup		Provider model without subgroup
	Subgroup 1	Subgroup 2	
Accuracy	0.898	0.875	0.901
Sensitivity	0.907	0.862	0.909
Precision	0.960	0.945	0.950
F_1_ score	0.932	0.901	0.928

### Feature Analysis

**Figure 13:**
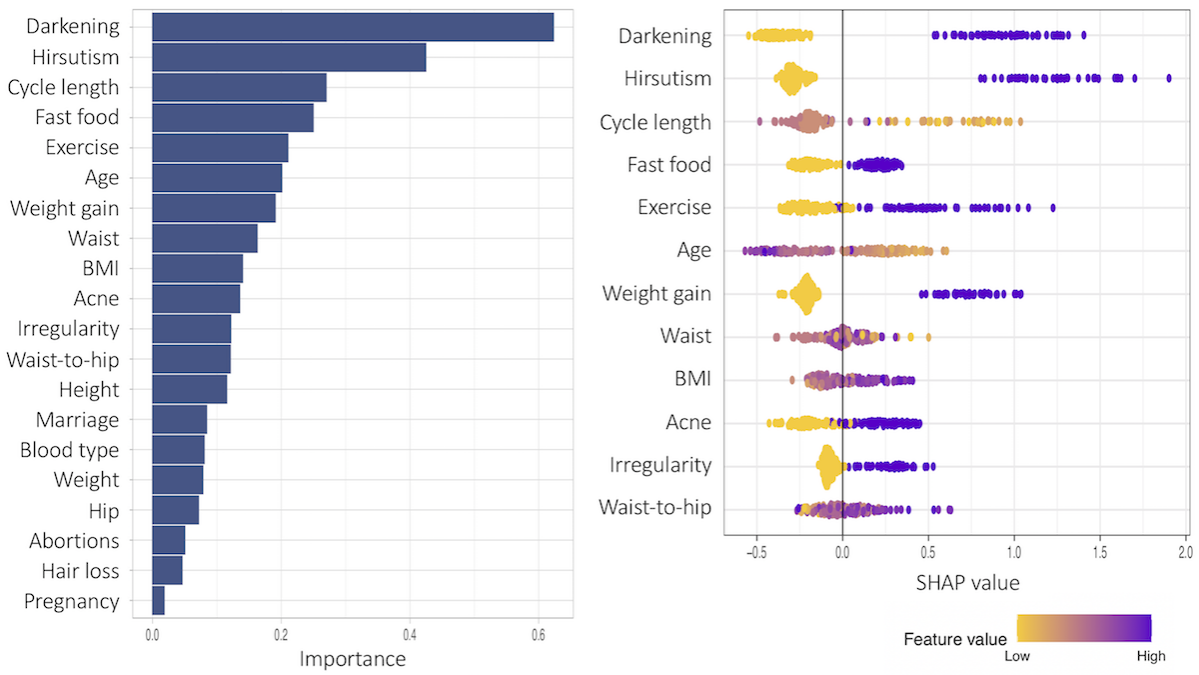
Feature importance for all variables and SHAP (Shapley Additive Explanations) values for the 12 most important features of subgroup 1 in the patient subgroup model including only noninvasive predictor variables.

**Figure 14:**
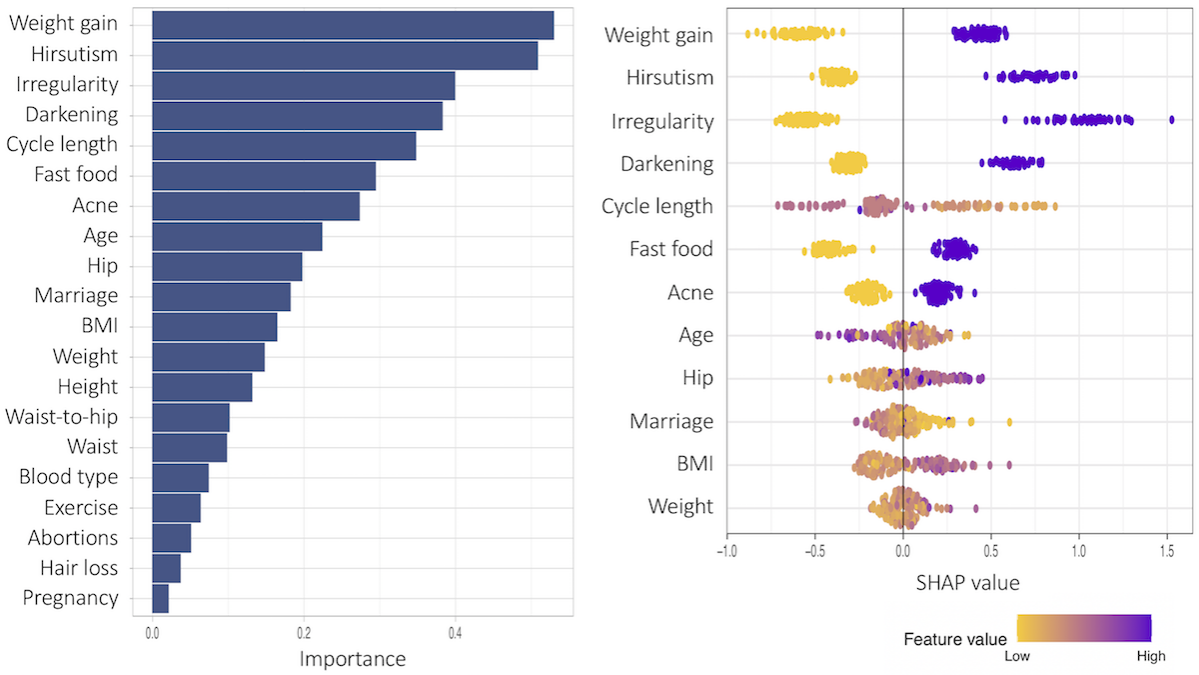
Feature importance for all variables and SHAP (Shapley Additive Explanations) values for the 12 most important features of subgroup 2 in the patient subgroup model including only noninvasive predictor variables.

**Figure 15:**
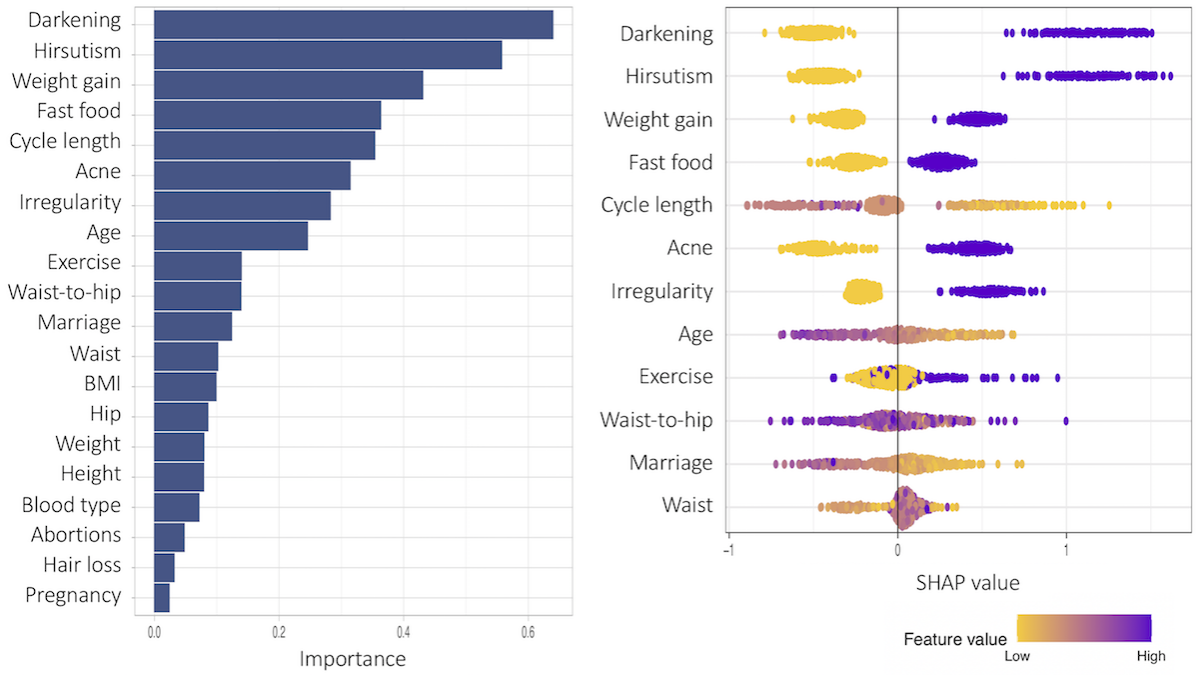
Feature importance for all variables (with the middle importance variables omitted) and the SHAP (Shapley Additive Explanations) values for the 12 most important features of the one to all model (without subgroups) in the patient model.

The SHAP values and the corresponding feature importance are examined in this subsection. First, we present patient models based only on noninvasive predictor variables. Figures 13-15 show the total variable importance in the first column for all noninvasive predictor variables, and the SHAP values for the 12 most important variables are graphed in the second column for the patient models. Figure 13 shows the result for subgroup 1 and Figure 14 shows the result for subgroup 2. In both subgroups, all symptom variables except hair loss and the two given variables, fast food consumption and age, are important features. The major difference between the subgroups is that acanthosis nigricans (*Darkening* in the plot) is the most important feature in subgroup 1, and weight gain is the most important feature in subgroup 2. Another interesting finding is that exercise is more important in subgroup 1, and irregular menstrual cycle is more prominent in subgroup 2 than in the other subgroup. In Figure 15, the results from one model to all (without subgrouping) in the patient model are presented. All symptom variables except hair loss and the two given variables, fast food consumption and age, have high importance values as well.

The SHAP values provide detailed local behavior in terms of feature importance. Most binary variables have distinct positive and negative SHAP values. For example, the SHAP values for the darkening variable, top-ranked in subgroup 1 in Figure 13, are clustered on the negative side (yellow dots in the plot) and on the positive side (purple dots in the plot), and these 2 clusters are far away from zero. The yellow dots represent lower values in the darkening variable (Darkening=No) and these variable values negatively affect the model output, that is, negative change in log odds in the model output, whereas the purple dots show higher values in the darkening variable (Darkening=Yes), and these variable values positively affect the model output. However, the higher feature values (Darkening=Yes) in purple have a stronger impact on the model output than the lower feature values (Darkening=No) because the purple cluster is farther away from zero than the yellow cluster.

**Figure 16:**
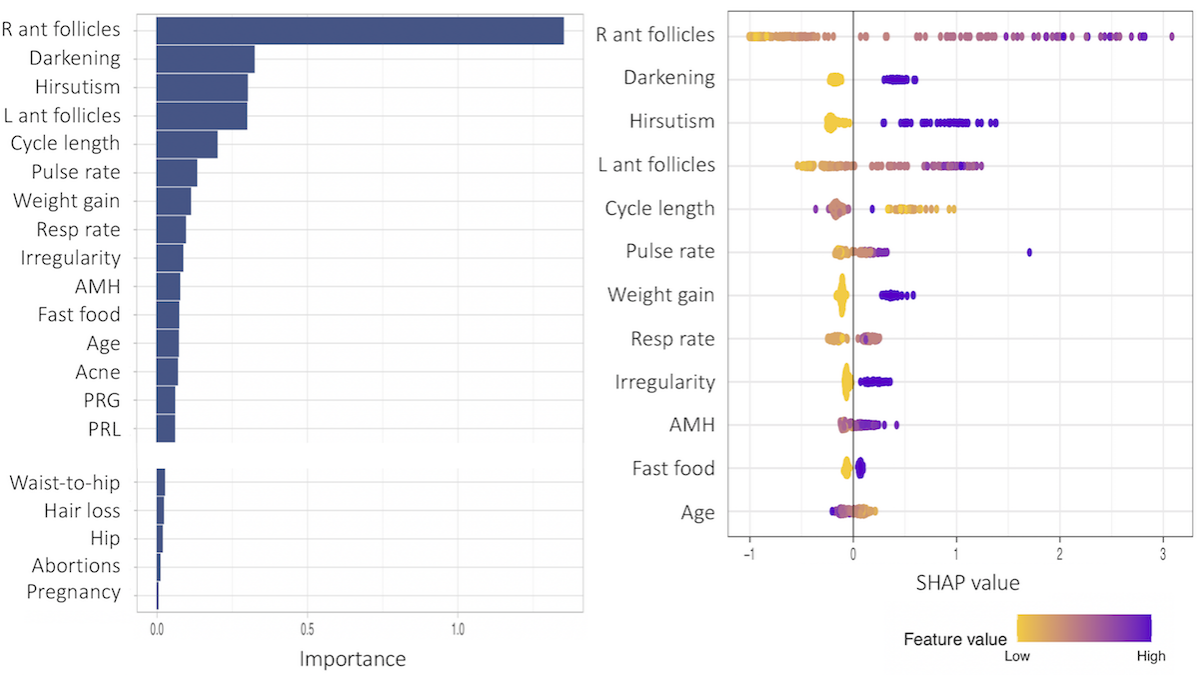
Feature importance for all variables (with the middle importance variables omitted) and SHAP (Shapley Additive Explanations) values for the 12 most important features of subgroup 1 in the provider subgroup model including all predictor variables. AMH: anti-Müllerian hormone; PRG: progrestrone; PRL: prolactin.

**Figure 17:**
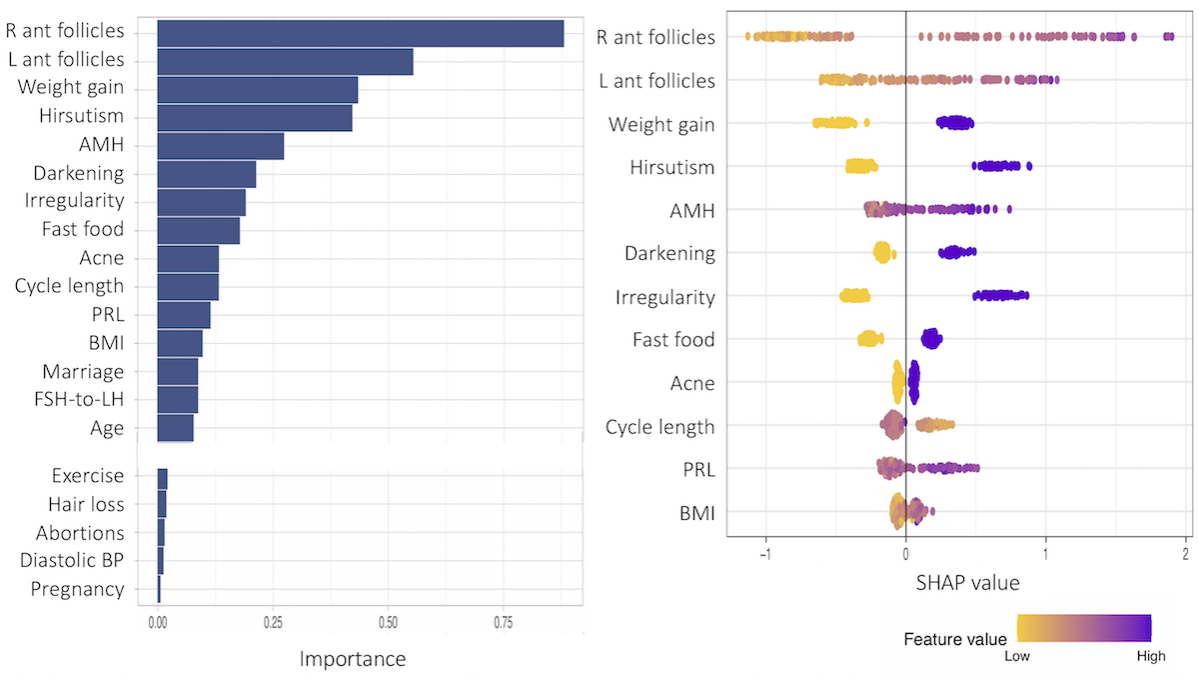
Feature importance for all variables (with the middle importance variables omitted) and SHAP (Shapley Additive Explanations) values for the 12 most important features of subgroup 2 in the provider subgroup model including all predictor variables. AMH: anti-Müllerian hormone; BP: blood pressure; FSH: follicle-stimulating hormone; LH: luteinizing hormone; PRL: prolactin.

**Figure 18:**
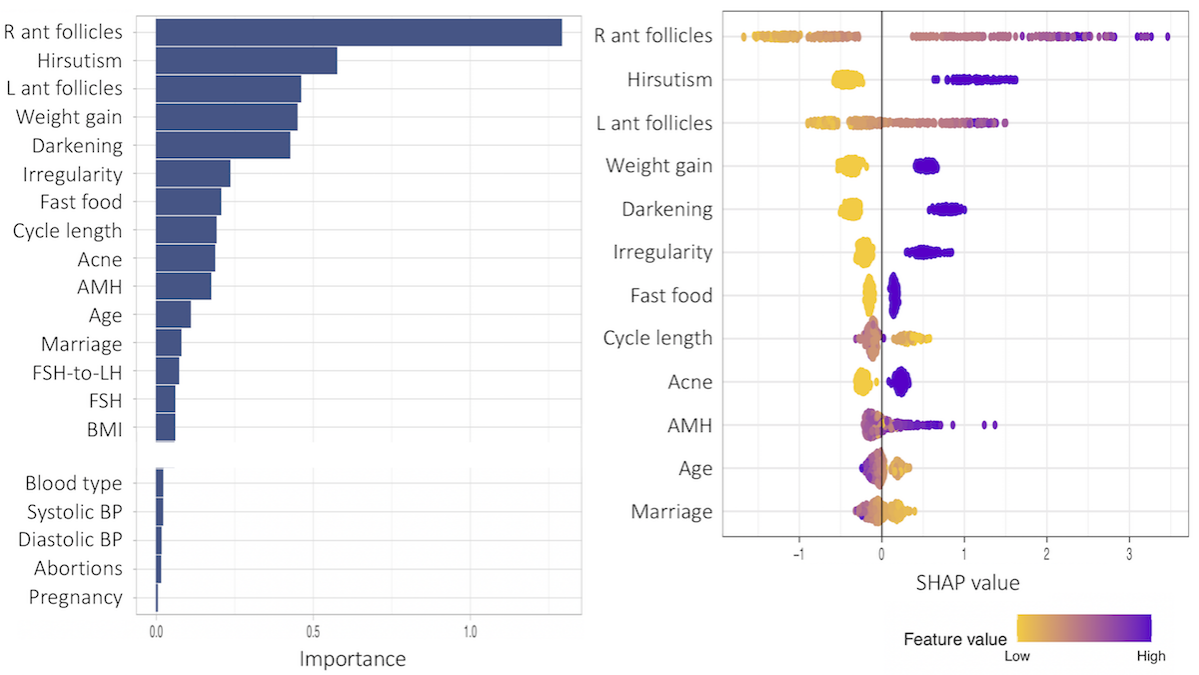
Feature importance for all variables (with the middle importance variables omitted) and the SHAP (Shapley Additive Explanations) values for the 12 most important features of the one to all model (without subgroups) in the provider model. AMH: anti-Müllerian hormone; BP: blood pressure; FSH: follicle-stimulating hormone; LH: luteinizing hormone; PRL: prolactin.

In Figures 16-18, we repeat the same process in the provider models. In all 3 figures, the number of antral follicles in the right ovary was most highly ranked in terms of feature importance. Otherwise, the number of antral follicles in the left ovary, all symptom attributes except hair loss, fast food consumption, and AMH are relatively more important than other variables, which are commonly observed in all 3 models. In comparison between subgroup 1 and subgroup 2, pulse rate and respiratory rate are more important features in subgroup 1 in Figure 16, and AMH is a more important feature (also note that the SHAP values of AMH are more spread out) in subgroup 2 than in the other subgroup, as shown in Figure 17. Regarding pulse rate and respiratory rate being important in subgroup 1, heart rate variability in normal weight women with PCOS has been subject to debate in the literature [35,36].

## Discussion

### Principal Findings

PCOS is a common, chronic, yet underrecognized female hormone disorder. Owing to the complexity of the syndrome, identification and differential diagnosis remain challenging even with widely accepted criteria and tests. Another disparity comes from the gaps in early diagnosis, information, and accessible support that can help women to prevent or manage this life span syndrome more adequately.

The ultimate goal of this study was to develop a conveniently accessible digital platform for pre- or self-diagnosis of PCOS using machine learning techniques such as PCA, *k*-means clustering algorithm, and CatBoost classifier based on the health measures of 526 female subjects. PCA was adopted to extract features from highly correlated anthropomorphic variables. The 2-means clustering algorithm was used to classify the 526 women into 2 different subgroups based on the first 5 principal components, leading to 2 subgroups with distinct BMIs. The gradient boosting decision tree-based classifier, CatBoost model, was trained and tested, and the prediction (test) error rates were compared between models based on four different error metrics: accuracy, sensitivity, precision, and F_1_ score.

We developed 2 types of prediction models targeting different groups of users. One model is for potential patients or other users who do not have medical test results available (the patient model) and the other is for clinical providers or other users who have access to the patients’ medical records and test results (the provider model). In each model, we applied a subgroup study to obtain the detailed characteristics of the 2 subgroups in the analysis. For the patient models, the prediction accuracy ranged from 81% to 81.5% with subgroups and 82.5% without subgroups. For the provider models, the prediction accuracy ranged from 87.5% to 89.8% with subgroups and 90.1% without subgroups.

Feature importance was performed in each model based on the SHAP values and the corresponding total feature importance. In the patient models, all symptom variables other than hair loss, that is, acanthosis nigricans, acne, hirsutism, irregular menstrual cycle, length of menstrual cycle, and weight gain, were important along with fast food consumption and age. For subgroup 1 (with lower BMI), acanthosis nigricans was the strongest marker in the prediction of PCOS status. Exercise was also an important factor in subgroup 1. For subgroup 2 (with higher BMI), weight gain was the top-ranked important marker and irregular menstrual cycle was also more prominent than subgroup 1. For the provider models, the number of antral follicles in the right ovary, the number of antral follicles in the left ovary, and AMH were important in all models in addition to the listed important variables in the patient models: acanthosis nigricans, acne, hirsutism, irregular menstrual cycle, length of menstrual cycle, weight gain, fast food consumption, and age. Another interesting finding was that the pulse rate and respiratory rates were also highly ranked in subgroup 1, which consisted of normal or underweight women with a mean weight of 52.353 kg and a mean BMI of 21.862 kg/m^2^.

The proposed prediction models based on available health measures are expected to provide women with a simpler and quicker access to a pre- or self-diagnosis of PCOS and possibly provide the opportunity for users to be educated and informed effectively. Gibson-Helm et al [37,38] reported that many women experience a prolonged and frustrating diagnosis of PCOS because it requires multiple health care providers to evaluate and diagnose PCOS, based on their study of a large community-based national sample. Our proposed work will resolve the concerns of delayed diagnosis and improve health care access for women.

### Limitations and Future Works

The main limitation of this study is that the sample is from a very specific population in Kerala, India, which is a state along India’s tropical Malabar coast. In previous studies, women experienced variations in PCOS symptoms depending on culture and ethnicity [38-40]. Another limitation is that the data set was collected from multiple hospitals where slightly different criteria might have been used to diagnose patients with PCOS, causing higher error rates in prediction. Our future work will include collecting larger data sets from different cultures and ethnicities for comparison and improving our prediction models. This will enable us to provide more tailored prediction tools for users in different cultural and ethnic groups. In addition, there is abundant evidence that PCOS substantially contributes to women’s anxiety disorders, depression, personality, and other psychological disorders [41,42]. A more in-depth study on the psychological effects of PCOS will be conducted in the future.

### Conclusions

In summary, the proposed study offers great potential that our self-diagnostic prediction models for PCOS status can serve as a convenient and easy-to-use digital platform based on available health measures for both potential patients and clinical providers.

## Abbreviations

AMH: anti-Müllerian hormone
BP: blood pressure
PCA: Principal Component Analysis
PCOS: polycystic ovary syndrome
SHAP: Shapley Additive Explanations
